# Prevalence and clinical course of upper airway respiratory virus infection in critically ill patients with hematologic malignancies

**DOI:** 10.1371/journal.pone.0260741

**Published:** 2021-12-14

**Authors:** Jongmin Lee, Seok Chan Kim, Chin Kook Rhee, Jaewoong Lee, Jong Wook Lee, Dong-Gun Lee

**Affiliations:** 1 Division of Pulmonary and Critical Care Medicine, Department of Internal Medicine, College of Medicine, The Catholic University of Korea, Seoul, Republic of Korea; 2 Department of Laboratory Medicine, Catholic Genetic Laboratory Center, College of Medicine, The Catholic University of Korea, Seoul, Republic of Korea; 3 Catholic Hematology Hospital, College of Medicine, The Catholic University of Korea, Seoul, Republic of Korea; 4 Divison of Infectious Diseases, Department of Internal Medicine, College of Medicine, The Catholic University of Korea, Seoul, Republic of Korea; University of Kentucky, UNITED STATES

## Abstract

**Background:**

The clinical significance of upper airway respiratory virus (RV) detection in patients with hematologic malignancies remains unclear. We aimed to investigate the association between upper airway RV detection and prognosis in critically ill patients with hematologic malignancies.

**Methods:**

This retrospective observational study included 331 critically ill patients with hematologic malignancies who presented respiratory symptoms and their nasopharyngeal swab was tested using a multiplex PCR assay between January 2017 and December 2018. A logistic regression model was used to adjust for potential confounding factors in the association between assay positivity and in-hospital mortality.

**Results:**

Among the 331 analyzed patients, RVs were detected in 29.0%. The overall mortality rates in the intensive care unit and hospital were 56.8% and 65.9%, respectively. Positive upper airway RV detection was associated with relapsed hematologic malignancies, higher level of C-reactive protein, and prior use of high dose steroids and anti-cancer chemotherapeutic drugs. Furthermore, it was independently associated with in-hospital mortality (adjusted odds ratio, 2.36; 95% confidence interval, 1.23 to 4.54). Among different RVs, parainfluenza virus was more prevalent among patients who died in the hospital than among those who survived (11.5% *vs*. 3.5%, *P* = 0.027).

**Conclusions:**

RV detection in the upper respiratory tract was relatively common in our cohort and was significantly associated with a poor prognosis. Thus, it can be used as a predictor of prognosis. Moreover, RV presence in the upper respiratory tract should be examined in patients who have previously been prescribed with high dose corticosteroids and anti-cancer drugs.

## Introduction

Acute respiratory failure (ARF) is known as the most common and life-threatening reason for intensive care unit (ICU) admission in critically ill patients with hematologic malignancies [1, 2]. The causes of ARF are various, including pulmonary infections, complications of anti-cancer chemotherapy, or pulmonary involvement of the malignancies [3–5]. Despite using comprehensive diagnostic workup, the etiology of ARF remains undetermined in up to 25% of the cases [3, 6].

Respiratory viruses (RVs), including the influenza virus, parainfluenza virus (PIV), respiratory syncytial virus (RSV), adenovirus (ADV), bocavirus, human metapneumovirus (MPV), coronavirus, and human rhinoviruses (HRV), are detected in up to 20% of ARF episodes in critically ill patients with cancer [6, 7]. In immunocompetent patients, RVs generally cause self-limited upper respiratory tract infections (URTI). However, RVs are associated with poor prognosis in immunocompromised hosts [8–12]. Traditionally, viral culture has been considered the gold standard for diagnosing RV infection [13]. However, real-time PCR has replaced the role of viral culture. In particular, new multiplex PCR tests now enable the rapid detection of a wide spectrum of RVs [14, 15].

Although previous studies have reported that up to 40% of patients with hematologic malignancies have one or more viruses detected in their upper respiratory tract (URT) samples, RVs have been much less considered as the cause of ARF [16–18]. A positive RV PCR test on URT may indicate asymptomatic carriage; however, it may also reflect true upper or lower respiratory tract infection [17–19].

To date, the clinical significance of upper airway RV detection in patients with hematologic malignancies remains unclear. Thus, our objective was to investigate the clinical impact of positive PCR test for RV presence in the URT in critically ill patients with hematologic malignancies and pneumonia.

## Materials and methods

### Patients

We retrospectively studied a cohort of critically ill patients with hematologic malignancies who were admitted to the medical and hematology ICUs of Seoul St. Mary’s Hospital (Seoul, Korea) between January 2017 and December 2018. In this hospital, more than 500 hematopoietic stem cell transplantations (HSCTs) are performed annually. The study population included patients with hematologic malignancies and respiratory symptoms who underwent testing for RVs in URT samples within 24 hours after ICU admission. Patients with active hematologic malignancies were defined as those undergoing concurrent evaluation and treatment without a diagnosis of remission [20]. Patients were identified by medical record review. This study was approved by the institutional review board of Seoul St. Mary’s Hospital (KC20RISI0297) and the requirement for informed consent was waived.

### Data collection

Epidemiologic and clinical data were collected from the patients’ medical charts at ICU admission. Data included sex; age; laboratory findings, including absolute neutrophil count (ANC), absolute lymphocyte count (ALC), high sensitivity C-reactive protein (hs-CRP), and procalcitonin level at ICU admission; characteristics of the hematologic malignancies, including the type of malignancy, current status, and prior treatments; presence of pneumonia at ICU admission; Sequential Organ Failure Assessment (SOFA) score at admission; use of corticosteroids, immunosuppressants, and anti-cancer chemotherapeutic drugs during the 30 days prior to ICU admission; use of mechanical ventilator; and the reason for ICU admission, including ARF, sepsis, and septic shock. Results of sputum cultures from appropriately collected specimens, bronchioalveolar lavage (BAL) fluid culture, BAL galactomannan test, Special stain (Gomori methenamine silver, Periodic Acid Schiff, or Ziehl-Neelsen) of BAL cells, atypical pneumonia serological panel (*Chlamydia*, *Legionella* and *Mycoplasma*), and serum galactomannan were also reviewed. Furthermore, comorbidity data were collected from the studied patients and used to calculate the Charlson comorbidity score index (CCI) [21].

Pneumonia was defined as the presence of a new infiltrate on a chest radiograph plus one or more of the following symptoms: fever (temperature ≥38.0 ˚C) or hypothermia (temperature <35.0 ˚C), new cough with or without sputum production, pleuritic chest pain, dyspnea, and altered breath sounds on auscultation [22]. The doses of corticosteroids were expressed as the prednisolone-equivalent doses. Respiratory symptoms included cough, sputum, and breathlessness. ARF was defined as oxygen saturation <90% or PaO_2_ <60 mmHg on room air, combined with a respiratory rate >30 breaths/min and/or clinical signs of respiratory distress [1, 23]. Sepsis and septic shock diagnosis were retrospectively confirmed based on medical records and according to international consensus guidelines [24]. Invasive fungal diseases including pulmonary aspergillosis (IPA) was defined with the presence of a compatible host factors, clinical features, and mycological evidence according to European Organization for Research and Treatment of Cancer/Mycoses Study Group criteria [25]. The diagnosis of *Pneumocystis jirovecii* pneumonia was based on the identification of the organism and/or PCR in BAL fluid with compatible clinical features and radiological findings [25]. Patients with nosocomial RV infection were defined as those admitted to the hospital for a reason other than acute respiratory infection, in whom respiratory symptoms developed ≥72 hours after admission, and in whom RV infection was confirmed using PCR [26]. Finally, we studied the clinical outcomes, including ICU and in-hospital mortality.

### Molecular assay for respiratory virus detection

Critically ill patients with hematologic malignancies and respiratory symptoms were screened for RVs at the discretion of physicians within 24 hours of admission; the clinical indications for testing included symptoms of an URTI or lower respiratory tract infection. Specimens for diagnostic testing included nasal swabs and nasopharyngeal aspirates. The RV PCR multiplex panel (AdvanSure RV real-Time PCR Kit; LG Life Sciences, Seoul, Korea) was used to test for influenza A and B viruses, PIV, ADV, RSV, MPV, HRV, bocavirus, and coronavirus. Nucleic acid extraction was performed using the QIAamp DNA Mini Kit in an automated extractor (Qiacube, Qiagen, Hilden, Germany).

### Statistical analysis

All results are reported as means ± standard deviations for normally distributed continuous variables and as medians and interquartile ranges for non-normally distributed continuous data. Categorical data are described as numbers (%). Patient characteristics were compared using the chi-squared test or Fisher’s exact test, as appropriate, for categorical variables, and independent samples *t*-tests for continuous variables. Multivariate analysis was performed to investigate associations between patient characteristics and hospital mortality. Odds ratios (OR) and the corresponding 95% confidence interval (CI) were computed. Goodness-of-fit was computed to assess the relevance of the logistic regression model. Probabilities of survival after ICU admission for each group were estimated using the Kaplan-Meier method and compared using the log rank test. All tests were two-sided, and *P* values <0.05 were considered statistically significant. All statistical analyses were performed using R 3.6.1 version (R Foundation, Vienna, Austria).

## Results

**Fig 1:**
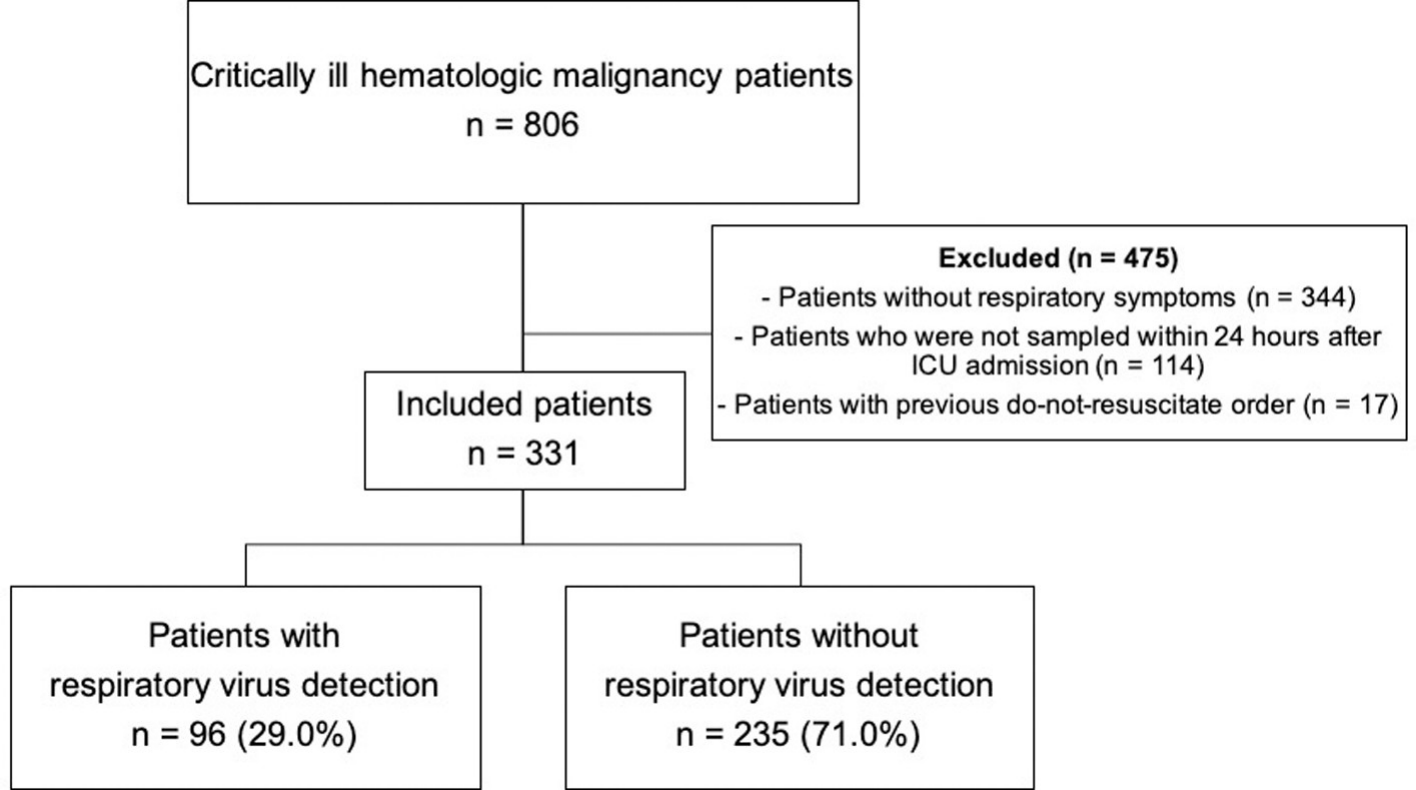
Study flow diagram. ICU, intensive care unit.

Among the 806 patients with hematologic malignancies admitted to our medical and hematology ICUs from January 2017 to December 2018, 331 (41.1%) had RV multiplex PCR test on their URT samples within 24 hours after ICU admission. Of these 331 patients, 96 (29.0%) had one or more positive RV PCR result (Fig 1).

### Patient characteristics

Table 1 shows the patients’ baseline characteristics. Of the 331 analyzed patients, 195 (58.9%) were men. The patients’ median age was 57.0 years. Almost half of the patients had acute leukemia (50.2%), 272 (82.2%) patients had active hematologic malignancies, 63 (19.0%) had relapsed diseases. and 104 (31.4%) were allogenic HSCT recipients. Pneumonia was present in 272 patients (82.2%) at ICU admission. ARF was present in 268 (81.0%) patients, and sepsis or septic shock were present in 272 (82.2%) patients. High flow nasal cannula was applied to 139 (42.0%) patients, and invasive mechanical ventilation was provided to 212 (64.1%) patients. Moreover, 101 (30.5%) patients received renal replacement therapy (RRT), while only 2 (0.6%) patients received extracorporeal membrane oxygenation therapy. The median SOFA score at ICU admission and the median CCI score were 9.0 (6.0–12.0) and 3.0 (2.0–5.0), respectively.

**Table 1:** Baseline characteristics of patients.

Variable		No. of patients (%) or median (IQR)
Sex, male		195 (58.9)
Age, yr		57.0 (44.0–67.0)
Underlying malignancies		
	Acute myeloid leukemia	118 (35.6)
	Acute lymphoblastic leukemia	48 (14.5)
	Chronic myeloid leukemia	12 (3.6)
	Multiple myeloma	53 (16.0)
	Myelodysplastic syndrome	22 (6.6)
	Lymphoma	61 (18.4)
	Others*	17 (5.1)
Malignancy state at ICU admission		
	Active disease	272 (82.2)
	Relapsed	63 (19.0)
	Complete remission	59 (17.8)
HSCT recipients		109 (32.9)
Autologous HSCT		19 (5.7)
Allogenic HSCT		104 (31.4)
Chronic respiratory insufficiency		35 (10.6)
Presence of pneumonia at ICU admission		272 (82.2)
Reasons for ICU admission		
Acute respiratory failure		268 (81.0)
Sepsis/Septic shock		272 (82.2)
Life-supporting interventions		
High flow nasal cannula		139 (42.0)
Mechanical ventilation		212 (64.0)
Renal replacement therapy		101 (30.5)
Extracorporeal membrane oxygenation		2 (0.6)
SOFA score at ICU admission		9.0 (6.0–12.0)
Charlson comorbidity index		3.0 (2.0–5.0)

No., number; IQR, interquartile range; HSCT, hematopoietic stem cell transplant; ICU, intensive care unit; SOFA, sequential organ failure assessment score.

*Others include primary myelofibrosis (5), hemophagocytic lymphohistiocytosis (1), Castleman disease (2), aplastic anemia (1), essential thrombocytosis (1), Waldenstrom macroglobulinemia (1), plasma cell leukemia (1), chronic lymphocytic leukemia (4), chronic eosinophilic leukemia (1).

### Respiratory virus and other pathogen detection

S1 Fig shows the distribution of patients according to the RV detected in URT samples by multiplex PCR. Overall, the most prevalent virus was PIV (30.2%, 29 of 96 patients with positive results), followed by HRV (22.9%, 22 of 96), RSV (14.6%, 14 of 96), coronavirus (13.5%, 13 of 96), influenza A and B viruses (8.3%, 8 of 96), MPV (7.3%, 7 of 96), ADV (5.2%, 5 of 96), and bocavirus (1.0%, 1 of 96). Only 3.1% of the patients (3 of 96) with positive RV PCR results had coinfections by two or more viruses. S1 Table and S2 Fig show the seasonal distribution of RV cases for each type of virus. Influenza viruses and RSV were significantly more predominant in winter (*P* <0.001 for influenza, *P* = 0.031 for RSV), while PIV was more frequent during summer and autumn (*P* = 0.009). S2 Table shows the comparison of the percentages of patients with positive multiplex PCR results for RVs in general and for each respiratory pathogen according to presence of ARF and in-hospital mortality. Overall, RVs were more prevalent in patients with than without ARF (31.7% *vs*. 17.5%, *P* = 0.037) and in patients who died in the hospital than in those who survived (34.4% *vs*. 18.6%, *P* = 0.004). Among RVs, particularly PIV was more frequently detected in patients who died in the hospital than in survivors (11.5% *vs*. 3.5%, *P* = 0.027).

Appropriate respiratory specimen was collected in 238 patients out of a total of 331 patients. Of these 238 patients, bacteria or fungus were detected in 116 patients (S3 Table). Bacteria were detected in 84 (72.4%) patients, and among them, Acinetobacter baumannii was the most detected pathogen. Fungus were detected in 34 (29.3%) patients and Aspergillus species were the most common.

### Characteristics and outcomes in patients with and without upper airway respiratory virus infection

**Fig 2:**
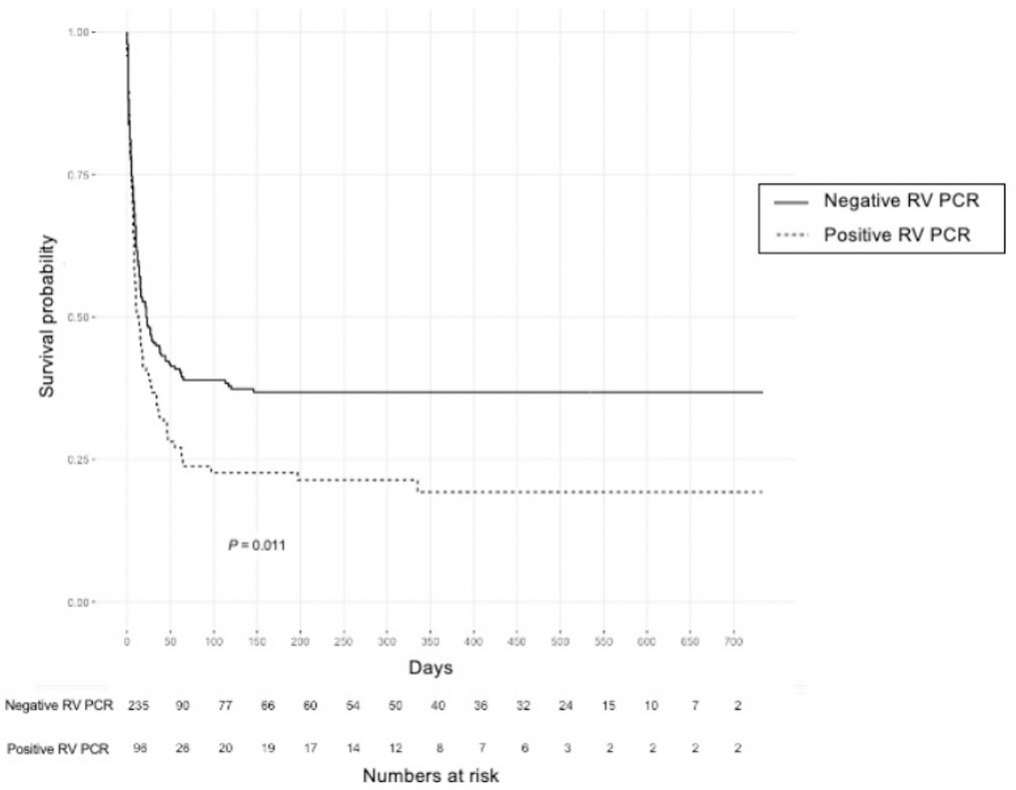
Kaplan-Meier curves of the probability of survival in patients with upper airway respiratory virus (positive RV PCR group, dotted line) and those without (negative RV PCR group, solid line). RV, respiratory virus.

Table 2 shows a comparison of the characteristics and outcomes between patients with positive and those with negative results in RV assay. The percentage of patients with relapsed hematologic malignancies was higher among patients with positive RV results than those with negative (30.2% *vs*. 14.5%, *P* = 0.002). The median level of hs-CRP was significantly higher in patients with positive than with negative RV PCR results (16.2 *vs*. 11.2, *P* = 0.027), but there was no difference in the level of procalcitonin between the two groups. The proportion of patients with ARF was significantly higher in patients with positive than with negative RV PCR results (88.5% *vs*. 77.9%, *P* = 0.037). The proportion of patients with sepsis or septic shock was not significantly different between the two groups. Moreover, the proportion of patients who used corticosteroids or anti-cancer chemotherapeutic drugs within 30 days prior to ICU admission was higher among patients with than those without upper airway RVs (corticosteroids: 87.5% *vs*. 63.8%, *P* < 0.001; chemotherapy: 68.8% *vs*. 55.7%, *P* = 0.039). Invasive mechanical ventilation was used significantly more often in RV-positive than RV-negative patients (74.0% *vs*. 60.0%, *P* = 0.023). ICU and in-hospital mortality rates were significantly higher in RV-positive than RV-negative patients (ICU: 68.8% *vs*. 51.9%, *P* = 0.007; hospital, 78.1% *vs*. 60.9%, *P* = 0.004). The probability of survival was significantly lower for patients positive for upper airway tract RV than those negative (*P* = 0.011, log-rank test; Fig 2).

**Table 2:** Comparison of clinical characteristics of patients with negative and positive upper airway respiratory virus PCR.

Variable	Negative RV PCR (n = 235)	Positive RV PCR (n = 96)	*P* value
Age, years	58.0 (44.0–67.5)	55.0 (43.5–64.0)	0.360
Sex, male	134 (57.0)	61 (63.5)	0.332
Underlying hematologic malignancies			
Acute myeloid leukemia	93 (39.6)	25 (26.0)	0.068
Acute lymphoblastic leukemia	32 (13.6)	15 (15.6)	
Chronic myeloid leukemia	11 (4.7)	1 (1.0)	
Multiple myeloma	31 (13.2)	22 (22.9)	
Myelodysplastic syndromes	16 (6.8)	6 (6.2)	
Lymphoma	39 (16.6)	22 (22.9)	
Others	13 (5.5)	5 (5.2)	
HSCT recipients			
Autologous HSCT	11 (4.7)	8 (8.3)	0.300
Allogenic HSCT	68 (28.9)	36 (37.5)	0.164
Disease status			
Active	192 (81.7)	80 (83.3)	0.846
Relapsed	34 (14.5)	29 (30.2)	0.002
SOFA score	9.0 (6.0–12.0)	10.0 (6.5–13.0)	0.135
Charlson Comorbidity Index	3.0 (2.0–5.0)	3.0 (2.0–4.0)	0.165
Presence of pneumonia on ICU admission	188 (80.0)	84 (87.5)	0.144
Laboratory findings on ICU admission			
Absolute neutrophil count, 10^9^/L	3.7 (0.1–7.4)	2.4 (0.2–6.8)	0.697
Absolute lymphocyte count, 10^9^/L	0.7 (0.2–1.7)	0.5 (0.1–1.3)	0.071
High sensitivity C-reactive protein, mg/dL	11.2 (5.4–22.1)	16.2 (9.3–23.5)	0.027
Procalcitonin, ng/mL	1.7 (0.3–10.6)	1.9 (0.9–5.0)	0.428
Season at ICU admission			0.419
Spring	62 (26.4)	19 (19.8)	
Summer	62 (26.4)	29 (30.2)	
Fall	61 (26.0)	22 (22.9)	
Winter	50 (21.3)	26 (27.1)	
Reasons for ICU admission			
Acute respiratory failure	183 (77.9)	85 (88.5)	0.037
Sepsis/Septic shock	194 (82.6)	78 (81.2)	0.902
Use of medications within 30 days prior to ICU admission			
Use of corticosteroids	150 (63.8)	84 (87.5)	<0.001
Accumulative prednisolone-equivalent dose, mg/kg	5.4 (0.0–15.4)	11.8 (4.2–21.8)	<0.001
Use of immunosuppressants	48 (20.4)	28 (29.2)	0.116
Use of anti-cancer chemotherapeutic drugs	131 (55.7)	66 (68.8)	0.039
Life-supporting interventions			
High flow nasal cannula	97 (41.3)	42 (43.8)	0.771
Mechanical ventilation	141 (60.0)	71 (74.0)	0.023
Renal replacement therapy	73 (31.1)	28 (29.2)	0.835
Extracorporeal membrane oxygenation	0 (0.0)	2 (2.1)	0.150
Prognosis of patients			
ICU mortality	122 (51.9)	66 (68.8)	0.007
In-hospital mortality	143 (60.9)	75 (78.1)	0.004

Data are presented as number (percentage) or as median (interquartile range).

PCR, polymerase chain reaction; HSCT, hematopoietic stem cell transplant; SOFA, sequential organ failure assessment score; ICU, intensive care u.

### Factors associated with positive upper airway respiratory virus detection among recipients of allogenic hematopoietic stem cell transplants

S4 Table shows a comparison of the characteristics and outcomes between patients with positive and those with negative RV PCR results among recipients and non-recipients of allogeneic HSCT. ALC was significantly lower in patients with positive than with negative upper airway RV results among allogeneic HSCT recipients (0.4 [0.2–1.1] *vs*. 0.9 [0.3–1.8] cells x 10^9^/L, *P* = 0.028). In both groups, steroid use within 30 days prior to ICU admission was significantly greater for RV-positive than RV-negative patients (allogeneic HSCT recipients: 94.4% *vs*. 72.1%, *P* = 0.014; non-recipients: 83.3% *vs*. 60.5%, *P* = 0.002). The doses of corticosteroids were also significantly greater among those with positive than those with negative RV PCR results (allogeneic HSCT recipients, 12.8 [5.3–23.8] *vs*. 6.9 [0.2–18.9] mg/kg prednisolone-equivalent dose, *P* = 0.026; non-recipients, 8.2 [2.0–19.1] *vs*. 5.3 [0.0–13.9] mg/kg prednisolone-equivalent dose, *P* = 0.010). In the allogeneic HSCT recipients’ group, in-hospital mortality rate was significantly higher among those with positive than with negative RV PCR results (83.3% *vs*. 61.8%, *P* = 0.041). Although ICU and in-hospital mortality rates were also higher among those with positive than with negative RV PCR results among non-HSCT recipients, the results were not statistically significant. S5 Table shows the comparison of patients’ distribution depending on positive multiplex PCR results for RVs in general and for each respiratory pathogen detected in URT samples between recipients and non-recipients of allogeneic HSCT. Among allogeneic HSCT recipients, PIV was the most common RV and significantly more prevalent than among non-recipients (15.4% *vs*. 5.7%, *P* = 0.007).

### Factors associated with increased hospital mortality

Univariate comparisons of the clinical characteristics of patients who survived and those who died are presented in S6 Table. Relapsed hematologic malignancies were more likely among non-survivors than among survivors (24.3% *vs*. 8.8%, *P* = 0.001). Among non-survivors, a higher proportion of patients had positive upper airway RV PCR results (34.4% *vs*. 18.6%, *P* = 0.004), pneumonia on ICU admission (91.7% *vs*. 63.7%, *P* < 0.001), IPA (14.2% *vs*. 6.2%, *P* = 0.047), ARF (85.3% *vs*. 72.6%, *P* = 0.008), and sepsis or septic shock (82.1% *vs*. 60.2%, *P* < 0.001) than the respective proportions of patients among survivors. Moreover, non-survivors had a higher SOFA score (11.0 [8.0–13.0] *vs*. 7.0 [4.0–9.0], *P* < 0.001), lower ANC (2.2 [0.0–6.8] *vs*. 4.5 [0.5–7.9] cells × 10^9^/L, *P* = 0.009), and higher hs-CRP (14.9 [7.7–24.4] *vs*. 8.8 [2.8–19.3] mg/dL, *P* < 0.001) than survivors, while more patients among non-survivors than among survivors required mechanical ventilation (81.7% *vs*. 30.1%, *P* < 0.001) and RRT (37.6% *vs*. 16.8%, *P* < 0.001). RV PCR positivity was independently associated with in-hospital mortality on multivariate analysis after adjusting for potential confounding factors (adjusted OR 2.36, 95% CI 1.23–4.54, *P* = 0.010; Table 3). Other independent risk factors for in-hospital mortality included relapsed hematologic malignancy (adjusted OR 2.63, 95% CI 1.16–6.00, *P* = 0.021), presence of pneumonia on ICU admission (adjusted OR 4.63, 95% CI 2.19–9.81, *P* < 0.001), requirement of RRT (adjusted OR 2.85, 95% CI 1.48–5.49, *P* = 0.002), and the presence of sepsis or septic shock on ICU admission (adjusted OR 2.33, 95% CI 1.24–4.36, *P* = 0.008). However, IPA was not independently associated with mortality after controlling for confounding factors (adjusted OR 1.52, 95% CI 0.57–4.09, *P* = 0.406).

**Table 3:** Clinical factors affecting hospital mortality.

Variable	Univariable			Multivariable		
	Crude OR	95% CI	*P* value	Adjusted OR	95% CI	*P* value
Positive upper airway RV PCR	2.30	1.33–3.98	0.003	2.36	1.23–4.54	0.010
Relapsed hematologic malignancy	3.31	1.61–6.79	0.001	2.63	1.16–6.00	0.021
Presence of pneumonia on ICU admission	6.33	3.42–11.72	<0.001	4.63	2.19–9.81	<0.001
Invasive pulmonary aspergillosis	2.51	1.07–5.90	0.035	1.52	0.57–4.09	0.406
High sensitivity C-reactive protein	1.04	1.01–1.06	0.002	1.02	0.99–1.05	0.116
Absolute neutrophil count on ICU admission	0.99	0.96–1.02	0.502	0.99	0.95–1.03	0.638
Renal replacement therapy	2.98	1.70–5.24	< 0.001	2.85	1.48–5.49	0.002
Acute respiratory failure	2.20	1.26–3.84	0.006	1.17	0.59–2.31	0.661
Sepsis/Septic shock on ICU admission	3.04	1.82–5.07	< 0.001	2.33	1.24–4.36	0.008

OR, odds ratio; CI, confidence interval; RV, respiratory virus; PCR, polymerase chain reaction; ICU, intensive care unit.

S7 Table shows the comparison of demographic and clinical characteristics between survivors and non-survivors among patients with positive RV PCR results. Notably, non-survivors were significantly older and had higher SOFA and CCI scores. A significantly larger proportion of patients had pneumonia at ICU admission (92.0% *vs*. 71.4%, *P* = 0.032) and required mechanical ventilation during ICU stay among non-survivors, compared to the respective proportions among survivors (86.7% *vs*. 28.6%, *P* < 0.001). The use of immunosuppressants, corticosteroids, and anti-cancer chemotherapeutic agents was not associated with mortality in patients with positive RV PCR results. After adjusting for potential confounding factors, in-hospital mortality remained significantly associated with the SOFA score (adjusted OR 1.20, 95% CI 1.11–1.31, *P* <0.001), the presence of pneumonia at ICU admission (adjusted OR 4.90, 95% CI 2.33–10.29, *P* <0.001), and the use of mechanical ventilation during ICU stay (adjusted OR 6.86, 95% CI 3.81–12.36, *P* <0.001) among patients with positive RV PCR results (S8 Table).

## Discussion

In this study, we aimed to evaluate the prevalence and impact of RV positivity in the upper airway tract of patients with hematologic malignancies. We found that the rates of in-hospital mortality and ARF was significantly higher among patients with positive than with negative upper airway RV PCR results. Furthermore, RV detection in the URT was relatively common among patients with hematologic malignancies and respiratory symptoms who required ICU admission.

In immunocompromised patients with respiratory failure, identification of ARF causes is important to improve the outcomes [27, 28]. Bacterial or fungal infections, including that by *Aspergillus* and *Pneumocystis jirovecii*, are usually considered to cause ARF in these patients, while RVs are rarely considered as the cause [16, 19]. RVs usually cause mild URTIs, but they sometimes cause severe ARF that requires life supporting interventions. Previous studies have reported that RVs are usually found in critically ill patients with pneumonia and associated with ARF and in-hospital mortality [29, 30]. Although RV PCR is recently used to identify the cause of ARF [31], the clinical relevance of RV detection in the URT in critically ill immunocompromised patients, such as those with hematologic malignancies, is still unclear.

In this study, RVs were detected in 29.0% of critically ill patients with hematologic malignancies and respiratory symptoms. This rate is lower than the rates reported in a few previous studies that included immunocompromised patients: 47% (47/100) among French immunocompromised critically ill patients [19] and 63% (33/52) among Dutch adult HSCT recipients with respiratory tract illness [18]. However, considering studies using tests on URT samples, our results are consistent with those of a previous multicenter prospective study, in which 26% of critically ill patients hematologic with malignancy were positive for RVs [30].

Upper airway tract RV detection was a predictor of ARF, and ICU and in-hospital mortality in the present study. In this study, IPA was not independently associated with in-hospital mortality after adjusting confounding factors. Although IPA is a well-known risk factor for mortality in critically ill patients with hematologic malignancies [32], our study revealed that respiratory viruses were independently associated with mortality. Previous studies have reported the important impact of RV infections on the morbidity and mortality of immunocompromised patients. For example, Fazekas *et al*. reported that viral URTI in immunocompromised children is associated with high morbidity, particularly among HSCT recipients [33]. Legoff *et al*. also reported that RV detection in the URT is associated with ARF and higher ICU mortality [30]. The findings of these studies are consistent with those in our study.

In this study, PIV was the most prevalent RV among those detected in the upper airway and was significantly associated with mortality. PIV infection can cause a variety of clinical syndromes, ranging from mild URTI to severe pneumonia [34]. Marcolini *et al*. previously reported that PIV infection is associated with high rates of pneumonia and mortality, as well as low ALC, in patients with hematologic malignancies [35]. Among immunocompromised patients in our study, HSCT recipients were the most affected by PIV infection. Using viral culture and direct immunofluorescence assay, infection rates of PIV have been shown to range from 2.2% to 7.1% among adult HSCT recipients [36–39]. In our study, the prevalence of PIV in allogeneic HSCT recipients was higher (15.4%) than that previously reported, possibly because of the high sensitivity of the multiplex RV PCR.

Consistently to previous studies, we found that the use and higher dose of corticosteroids are risk factors for positive RV detection in URT. This result was the same both for allogenic HSCT recipients and non-HSCT recipients. Systemic steroids have many effects on the innate and acquired immunity, predisposing patients to infection [40, 41]. A previous study also reported that prior treatment with corticosteroids or other immunosuppressants increases the risk for viral infection in immunocompromised patients [30]. Furthermore, in allogeneic HSCT recipients, we also found that lymphopenia is a risk factor for positive upper airway tract RV detection. Previous studies about general population have shown that lymphopenia is associated with increased infection rates and infection-related deaths [12, 42]. It has also been reported that lymphopenia is a risk factor for mortality among HSCT recipients with viral infection [43, 44]. It is likely that detection of RVs the in upper airway is associated with a decreased T-cell-mediated immune response after the administration of corticosteroids [45]. These data suggest that clinicians should use corticosteroids more cautiously in patients with hematologic malignancies.

The present study had several limitations. First, this study was not prospective and performed at a single center. Therefore, selection bias may have affected the significance of our findings. However, in this single large, 2-year cohort whose treatment was based on the same protocol, we carefully inspected all patients admitted to the ICU with respiratory symptoms and sampled for RV PCR within 24 hours after ICU admission. The aim of this study was to identify the clinical impact of upper airway RV in critically ill patients with hematologic malignancies; thus, the setting of our study did not greatly differ from that of a prospective observational study. Second, lower respiratory tract samples had not been collected for the patients in our study. Therefore, we cannot conclude whether RV positivity reflects true upper or lower respiratory tract infection. Nonetheless, positive nasopharyngeal RV PCR results were significantly associated with in-hospital mortality in this study. A possible explanation for this might be that positive PCR results are a marker for poor immune function in critically ill patients with hematologic malignancies. The presence of viruses in the upper airway may render the host epithelium more susceptible to bacterial colonization, and as a result, promote bacterial pneumonia [46]. Accordingly, previous studies have reported that viral coinfection is associated with a worse prognosis in patients with community-acquired bacterial pneumonia [47, 48]. Third, our study showed a relatively higher mortality rate (65.9%) compared to that reported by other studies regarding critically ill patients with hematologic malignancies (43.0%) [30]. This difference may be explained by the different enrollment criteria. In this study, we enrolled critically ill hematologic malignancy patients with respiratory symptoms, and ARF was the most common reason (81%) for ICU admission. Moreover, many patients had characteristics associated with poor prognosis, such as relapsed malignancies, which definitely contributed to the high mortality rate.

## Conclusion

We found that RV detection in the URT is relatively common and is significantly associated with poor prognosis in critically ill patients with hematologic malignancies and respiratory symptoms. Among RVs, PIV was more prevalent among patients who died in the hospital. Prior use and higher doses of corticosteroids were associated with positive upper airway RV PCR results. These results indicate that RV detection in the upper respiratory is a predictor of poor prognosis, while patients who have previously received high doses of corticosteroids should be screened for RVs by PCR. Further studies on the mechanistic analysis of critically ill patients with hematologic malignancies who are positive for RVs in the URT are warranted.

## Supporting information

S1 Fig Patients’ distribution according to the type of upper airway respiratory virus detected in nasopharyngeal samples by PCR. PIV, parainfluenza virus; RSV, respiratory syncytial virus; Boca, bocavirus; Corona, coronavirus; HRV, rhinovirus; MPV, metapneumovirus; ADV, adenovirus.
(TIF)

S2 Fig Monthly distribution of upper airway respiratory virus detection cases. PIV, parainfluenza virus; RSV, respiratory syncytial virus; Boca, bocavirus; Corona, coronavirus; HRV, rhinovirus; MPV, metapneumovirus; ADV, adenovirus.
(TIF)

S1 Table Seasonal distribution of respiratory virus detection cases. (DOCX)

S2 Table Comparison of upper airway RV PCR according to presence of acute respiratory failure and in-hospital mortality. (DOCX)

S3 Table Pathogens identified from respiratory specimens in patients (n = 116). (DOCX)

S4 Table Comparison of the clinical characteristics of allogenic hematopoietic stem cell transplant recipients and non-recipients with or without positive upper airway respiratory virus PCR. (DOCX)

S5 Table Comparison of proportions of patients positive for RVs by PCR between allogeneic hematopoietic stem cell transplant recipients and non-recipients. (DOCX)

S6 Table Comparison of the clinical characteristics of hospital survivors and non-survivors. (DOCX)

S7 Table Comparison of the clinical characteristics of hospital survivors and non-survivors in patients with positive upper airway respiratory virus PCR. (DOCX)

S8 Table Multivariate analyses with logistic regression models for probability of in-hospital mortality in patients with positive upper airway respiratory virus PCR. (DOCX)
