# Supplementary figures and images for: Prevalence and clinical course of upper airway respiratory virus infection in critically ill patients with hematologic malignancies
Source: PLoS One. 2021 Dec 14;16(12):e0260741. doi: 10.1371/journal.pone.0260741 (PMC8670702; doi:10.1371/journal.pone.0260741)

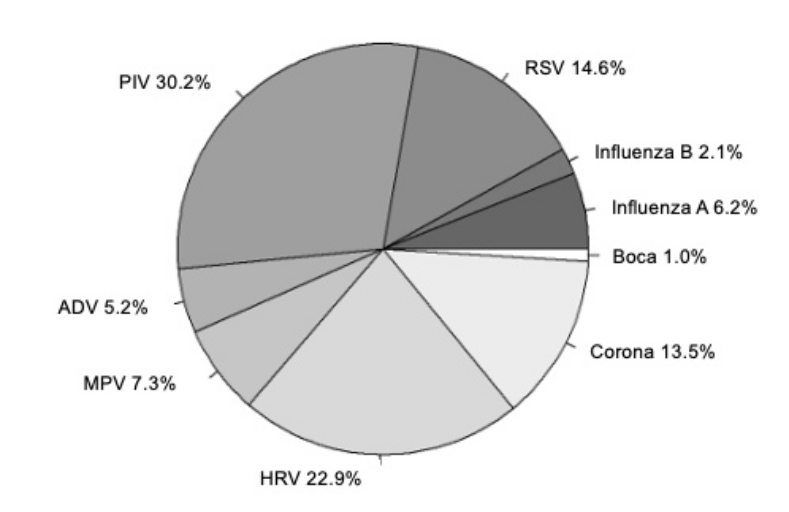

Supplement: S1 Fig — PIV, parainfluenza virus; RSV, respiratory syncytial virus; Boca, bocavirus; Corona, coronavirus; HRV, rhinovirus; MPV, metapneumovirus; ADV, adenovirus. (TIF) [file pone.0260741.s001.tif]

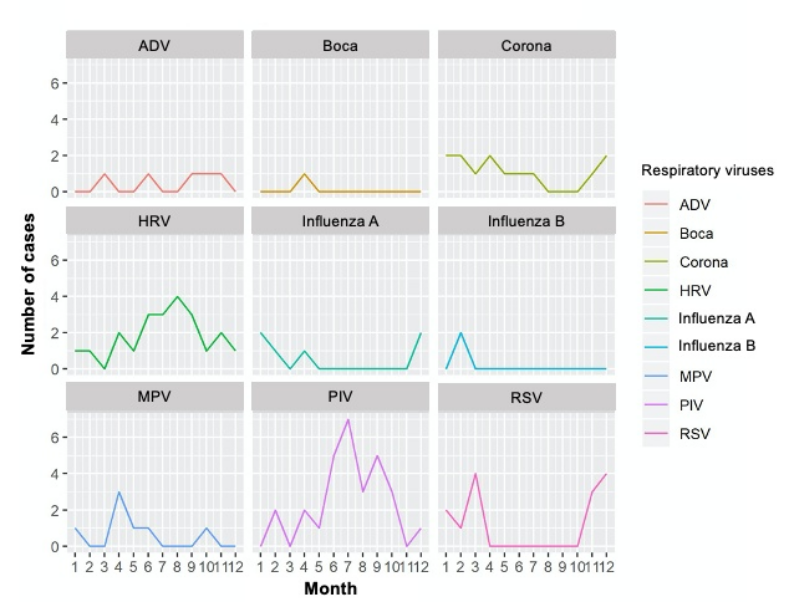

Supplement: S2 Fig — PIV, parainfluenza virus; RSV, respiratory syncytial virus; Boca, bocavirus; Corona, coronavirus; HRV, rhinovirus; MPV, metapneumovirus; ADV, adenovirus. (TIF) [file pone.0260741.s002.tif]
