# Supplementary material for: Prevalence and clinical course of upper airway respiratory virus infection in critically ill patients with hematologic malignancies
Source: PLoS One. 2021 Dec 14;16(12):e0260741. doi: 10.1371/journal.pone.0260741 (PMC8670702; doi:10.1371/journal.pone.0260741)
Supplement: S1 Table — (DOCX) [file pone.0260741.s003.docx]

**S1 Table. Seasonal distribution of respiratory virus detection cases**

| Variable | Spring^a^ | Summer^b^ | Autumn^c^ | Winter^d^ | *P* value |
| --- | --- | --- | --- | --- | --- |
| Positive upper airway RV PCR | 19 (23.5) | 29 (31.9) | 22 (26.5) | 26 (34.2) | 0.419 |
| Influenza A & B | 1 (1.2) | 0 (0.0) | 0 (0.0) | 7 (9.2) | <0.001 |
| Respiratory syncytial virus | 4 (4.9) | 0 (0.0) | 3 (3.6) | 7 (9.2) | 0.031 |
| Parainfluenza | 3 (3.7) | 15 (16.5) | 8 (9.6) | 3 (3.9) | 0.009 |
| Rhinovirus | 3 (3.7) | 10 (11.0) | 6 (7.2) | 3 (3.9) | 0.184 |
| Metapneumovirus | 4 (4.9) | 1 (1.1) | 1 (1.2) | 1 (1.3) | 0.247 |
| Adenovirus | 1 (1.2) | 1 (1.1) | 3 (3.6) | 0 (0.0) | 0.286 |
| Coronavirus | 4 (4.9) | 2 (2.2) | 1 (1.2) | 6 (7.9) | 0.125 |
| Bocavirus | 1 (1.2) | 0 (0.0) | 0 (0.0) | 0 (0.0) | 0.377 |

RV, respiratory virus; PCR, polymerase chain reaction

^a^ From March to May

^b^ From June to August

^c^ From September to November

^d^ From December to February
