# Supplementary material for: Prevalence and clinical course of upper airway respiratory virus infection in critically ill patients with hematologic malignancies
Source: PLoS One. 2021 Dec 14;16(12):e0260741. doi: 10.1371/journal.pone.0260741 (PMC8670702; doi:10.1371/journal.pone.0260741)
Supplement: S2 Table — (DOCX) [file pone.0260741.s004.docx]

**S2 Table. Comparison of upper airway RV PCR according to presence of acute respiratory failure and in-hospital mortality**

| Variable | Patients without ARF  (n = 63) | Patients with ARF  (n = 268) | *P* value | Survivors  (n = 113) | Patients who died  (n = 218) | *P* value |
| --- | --- | --- | --- | --- | --- | --- |
| Positive upper airway RV PCR | 11 (17.5) | 85 (31.7) | 0.037 | 21 (18.6) | 75 (34.4) | 0.004 |
| Influenza A & B | 0 (0.0) | 8 (3.0) | 0.351 | 3 (2.7) | 5 (2.3) | 1.000 |
| Respiratory syncytial virus | 3 (4.8) | 11 (4.1) | 1.000 | 3 (2.7) | 11 (5.0) | 0.461 |
| Parainfluenza | 3 (4.8) | 26 (9.7) | 0.317 | 4 (3.5) | 25 (11.5) | 0.027 |
| Rhinovirus | 2 (3.2) | 20 (7.5) | 0.343 | 9 (8.0) | 13 (6.0) | 0.645 |
| Metapneumovirus | 1 (1.6) | 6 (2.2) | 1.000 | 1 (0.9) | 6 (2.8) | 0.473 |
| Adenovirus | 1 (1.6) | 4 (1.5) | 1.000 | 0 (0.0) | 5 (2.3) | 0.251 |
| Coronavirus | 1 (1.6) | 12 (4.5) | 0.482 | 4 (3.5) | 9 (4.1) | 1.000 |
| Bocavirus | 1 (1.6) | 0 (0.0) | 0.430 | 0 (0.0) | 1 (0.5) | 1.000 |

RV, respiratory virus; PCR, polymerase chain reaction; ARF, acute respiratory failure
