# Supplementary material for: Prevalence and clinical course of upper airway respiratory virus infection in critically ill patients with hematologic malignancies
Source: PLoS One. 2021 Dec 14;16(12):e0260741. doi: 10.1371/journal.pone.0260741 (PMC8670702; doi:10.1371/journal.pone.0260741)
Supplement: S3 Table — (DOCX) [file pone.0260741.s005.docx]

**S3 Table. Pathogens identified from respiratory specimens in patients (n = 116).**

| Pathogen | No. (%) |
| --- | --- |
| Bacteria (n = 84) |  |
| *Acinetobacter baumannii* | 23 (27.4) |
| *Staphylococcus aureus* | 17 (20.2) |
| *Corynebacterium* species | 10 (11.9) |
| *Klebsiella* species | 8 (9.5) |
| *Pseudomonas aeruginosa* | 6 (7.1) |
| *Stenotrophomonas maltophilia* | 5 (6.0) |
| *Non-Tuberculosis mycobacterium* | 5 (6.0) |
| *Enterobacter* species | 3 (3.6) |
| *Escherichia coli* | 3 (3.6) |
| *Mycobacterium tuberculosis* | 2 (2.4) |
| Others^a^ | 3 (3.6) |
| Fungus (n = 34) |  |
| *Aspergillus* species | 22 (64.7) |
| *Pneumocystis jirovecii* | 10 (29.4) |
| *Hyaline hyphae* | 2 (5.9) |

^a^ Others: *Brevibacterium* species (1), *Chryseobacterium* species (2)
