# Supplementary material for: Prevalence and clinical course of upper airway respiratory virus infection in critically ill patients with hematologic malignancies
Source: PLoS One. 2021 Dec 14;16(12):e0260741. doi: 10.1371/journal.pone.0260741 (PMC8670702; doi:10.1371/journal.pone.0260741)
Supplement: S4 Table — (DOCX) [file pone.0260741.s006.docx]

**S4 Table. Comparison of the clinical characteristics of allogenic hematopoietic stem cell transplant recipients and non-recipients with or without positive upper airway respiratory virus PCR.**

| Variable | Patients with allogenic HSCT | | | Patients without allogenic HSCT | | |
| --- | --- | --- | --- | --- | --- | --- |
|  | Negative RV PCR  (n = 68) | Positive RV PCR  (n = 36) | *P* value | Negative RV PCR  (n = 167) | Positive RV PCR  (n = 60) | *P* value |
| Age | 48.5 (40.5 – 58.5) | 50.0 (38.5 – 61.0) | 0.715 | 59 (48.0 – 70.0) | 57.0 (46.5 – 67.5) | 0.354 |
| Sex, Male | 37 (54.4) | 24 (66.7) | 0.318 | 97 (58.1) | 37 (61.7) | 0.741 |
| Disease status |  |  |  |  |  |  |
| Active | 52 (76.5) | 23 (63.9) | 0.258 | 75 (44.9) | 17 (28.3) | 0.037 |
| Relapsed | 32 (47.1) | 21 (58.3) | 0.375 | 160 (95.8) | 59 (98.3) | 0.616 |
| SOFA score | 7.0 (5.0 – 9.5) | 9.0 (6.5 – 12.0) | 0.102 | 10.0 (6.5 – 13.0) | 11.0 (6.5 – 13.0) | 0.285 |
| Charlson Comorbidity Index | 3.0 (2.0 – 4.0) | 3.0 (2.0 – 4.0) | 0.729 | 3.0 (2.0 – 5.0) | 3.0 (2.0 – 5.0) | 0.262 |
| Presence of pneumonia on ICU admission | 54 (79.4) | 29 (80.6) | 1.000 | 134 (80.2) | 55 (91.7) | 0.067 |
| Laboratory findings on ICU admission |  |  |  |  |  |  |
| Absolute neutrophil count, × 10^9^/L | 5.3 (1.0 – 9.5) | 3.5 (0.3 – 7.2) | 0.258 | 2.5 (0.1 – 6.5) | 1.9 (0.2 – 5.7) | 0.981 |
| Absolute lymphocyte count, × 10^9^/L | 0.9 (0.3 – 1.8) | 0.4 (0.2 – 1.1) | 0.028 | 0.6 (0.2 – 1.7) | 0.5 (0.1 – 1.7) | 0.438 |
| Procalcitonin, ng/mL | 1.0 (0.3 – 7.8) | 1.7 (0.4 – 7.5) | 0.461 | 2.4 (0.3 – 12.2) | 2.0 (0.9 – 5.0) | 0.622 |
| High sensitivity C-reactive protein, mg/dL | 7.7 (2.9 – 17.2) | 13.3 (5.2 – 17.7) | 0.150 | 12.9 (6.6 – 24.6) | 19.5 (11.7 – 25.1) | 0.032 |
| Reasons for ICU admission |  |  |  |  |  |  |
| Acute respiratory failure | 54 (79.4) | 30 (83.3) | 0.825 | 129 (77.2) | 55 (91.7) | 0.024 |
| Sepsis/Septic shock | 46 (67.6) | 26 (72.2) | 0.683 | 131 (78.4) | 44 (73.3) | 0.530 |
| Use of medications within 30 days prior to ICU admission |  |  |  |  |  |  |
| Use of corticosteroids | 49 (72.1) | 34 (94.4) | 0.014 | 101 (60.5) | 50 (83.3) | 0.002 |
| Accumulative prednisolone-equivalent dose, mg/kg | 6.9 (0.2 – 18.9) | 12.8 (5.3 – 23.8) | 0.026 | 5.3 (0.0 – 13.9) | 8.2 (2.0 – 19.1) | 0.010 |
| Use of immunomodulatory drugs | 45 (66.2) | 26 (72.2) | 0.683 | 3 (1.8) | 2 (3.3) | 0.855 |
| Use of chemotherapeutic drugs | 27 (39.7) | 16 (44.4) | 0.797 | 104 (62.3) | 50 (83.3) | 0.005 |
| Life-supporting interventions |  |  |  |  |  |  |
| High flow nasal cannula | 30 (44.1) | 20 (55.6) | 0.366 | 67 (40.1) | 22 (36.7) | 0.752 |
| Mechanical ventilation | 45 (66.2) | 28 (77.8) | 0.315 | 96 (57.5) | 43 (71.7) | 0.075 |
| Renal replacement therapy | 19 (27.9) | 11 (30.6) | 0.958 | 54 (32.3) | 17 (28.3) | 0.681 |
| Prognosis of patients |  |  |  |  |  |  |
| ICU mortality | 36 (52.9) | 26 (72.2) | 0.090 | 86 (51.5) | 40 (66.7) | 0.061 |
| In-hospital mortality | 42 (61.8) | 30 (83.3) | 0.041 | 101 (60.5) | 45 (75.0) | 0.063 |

Data are presented as number (percentage) or as median (interquartile range)

HSCT, hematopoietic stem cell transplant; PCR, polymerase chain reaction; SOFA, sequential organ failure assessment score; ICU, intensive care unit
