# Supplementary material for: Prevalence and clinical course of upper airway respiratory virus infection in critically ill patients with hematologic malignancies
Source: PLoS One. 2021 Dec 14;16(12):e0260741. doi: 10.1371/journal.pone.0260741 (PMC8670702; doi:10.1371/journal.pone.0260741)
Supplement: S5 Table — (DOCX) [file pone.0260741.s007.docx]

**S5 Table. Comparison of proportions of patients positive for RVs by PCR between allogeneic hematopoietic stem cell transplant recipients and non-recipients.**

RV, respiratory virus; PCR, polymerase chain reaction; HSCT, hematopoietic stem cell transplant

| Variable | Patients with allogenic HSCT  (n = 104) | Patients without allogeneic HSCT  (n = 227) | *P* value |
| --- | --- | --- | --- |
| Positive upper airway RV PCR | 36 (34.6) | 60 (26.4) | 0.164 |
| Influenza A & B | 1 (1.0) | 7 (3.1) | 0.435 |
| Respiratory syncytial virus | 8 (7.7) | 6 (2.6) | 0.068 |
| Parainfluenza | 16 (15.4) | 13 (5.7) | 0.007 |
| Rhinovirus | 4 (3.8) | 18 (7.9) | 0.251 |
| Metapneumovirus | 3 (2.9) | 4 (1.8) | 0.805 |
| Adenovirus | 2 (1.9) | 3 (1.3) | 1.000 |
| Coronavirus | 3 (2.9) | 10 (4.4) | 0.722 |
| Bocavirus | 0 (0.0) | 1 (0.4) | 1.000 |
