# Supplementary material for: Prevalence and clinical course of upper airway respiratory virus infection in critically ill patients with hematologic malignancies
Source: PLoS One. 2021 Dec 14;16(12):e0260741. doi: 10.1371/journal.pone.0260741 (PMC8670702; doi:10.1371/journal.pone.0260741)
Supplement: S6 Table — (DOCX) [file pone.0260741.s008.docx]

**S6 Table. Comparison of the clinical characteristics of hospital survivors and non-survivors**

| Variable | Survived patients  (n = 113) | Died patients  (n = 218) | *P* value |
| --- | --- | --- | --- |
| Age | 54.0 (41.0 – 66.0) | 58.0 (46.0 – 67.0) | 0.099 |
| Sex, Male | 66 (58.4) | 129 (59.2) | 0.987 |
| Underlying hematologic malignancies |  |  |  |
| Acute myeloid leukemia | 40 (35.4) | 78 (35.8) | 0.072 |
| Acute lymphoblastic leukemia | 17 (15.0) | 30 (13.8) |  |
| Chronic myeloid leukemia | 5 (4.4) | 7 (3.2) |  |
| Multiple myeloma | 21 (18.6) | 32 (14.7) |  |
| Myelodysplastic syndromes | 5 (4.4) | 17 (7.8) |  |
| Lymphoma | 14 (12.4) | 47 (21.6) |  |
| Others | 11 (9.7) | 7 (3.2) |  |
| Allogenic HSCT recipients | 32 (28.3) | 72 (33.0) | 0.453 |
| Disease status |  |  |  |
| Active | 27 (23.9) | 32 (14.7) | 0.054 |
| Relapsed | 10 (8.8) | 53 (24.3) | 0.001 |
| Positive upper airway RV PCR | 21 (18.6) | 75 (34.4) | 0.004 |
| SOFA score on ICU admission | 7.0 (4.0 – 9.0) | 11.0 (8.0 – 13.0) | <0.001 |
| Charlson Comorbidity Index | 3.0 (2.0 – 4.0) | 3.0 (2.0 – 5.0) | 0.101 |
| Presence of pneumonia on ICU admission | 72 (63.7) | 200 (91.7) | <0.001 |
| Invasive pulmonary aspergillosis | 7 (6.2) | 31 (14.2) | 0.047 |
| Laboratory findings on ICU admission |  |  |  |
| Absolute neutrophil count, × 10^9^/L | 4.5 (0.5 – 7.9) | 2.2 (0.0 – 6.8) | 0.009 |
| Absolute lymphocyte count, × 10^9^/L | 0.7 (0.3 – 1.6) | 0.6 (0.1 – 1.8) | 0.492 |
| Procalcitonin, ng/mL | 1.4 (0.2 – 11.4) | 2.0 (0.6 – 8.6) | 0.406 |
| High sensitivity C-reactive protein, mg/dL | 8.8 (2.8 – 19.3) | 14.9 (7.7 – 24.4) | <0.001 |
| Reasons for ICU admission |  |  |  |
| Acute respiratory failure | 82 (72.6) | 186 (85.3) | 0.008 |
| Sepsis/Septic shock | 68 (60.2) | 179 (82.1) | <0.001 |
| Life-supporting interventions |  |  |  |
| High flow nasal cannula | 41 (36.3) | 98 (45.0) | 0.162 |
| Mechanical ventilation | 34 (30.1) | 178 (81.7) | <0.001 |
| Renal replacement therapy | 19 (16.8) | 82 (37.6) | <0.001 |
| Extracorporeal membrane oxygenation | 0 (0.0) | 2 (0.9) | 0.785 |
| Use of medications in 30 days prior to ICU admission |  |  |  |
| Use of corticosteroids | 79 (69.9) | 155 (71.1) | 0.922 |
| Accumulative prednisolone-equivalent dose, mg/kg | 5.7 (0.0 – 14.5) | 7.4 (0.0 – 18.7) | 0.513 |
| Use of immunomodulatory drugs | 23 (20.4) | 53 (24.3) | 0.500 |
| Use of chemotherapeutic drugs | 60 (53.1) | 137 (62.8) | 0.111 |
| Other pathogens identified from respiratory specimens (n = 238)^a^ | 30 (41.1) | 86 (52.1) | 0.153 |
| Bacteria | 19 (26.0) | 65 (39.4) | 0.065 |
| Fungus | 12 (16.4) | 22 (13.3) | 0.667 |

Data are presented as number (percentage) or as median (interquartile range)

HSCT, hematopoietic stem cell transplantation; RV, respiratory virus; PCR, polymerase chain reaction; SOFA, sequential organ failure assessment score; ICU, intensive care unit

^a^Appropriate lower respiratory tract specimen was collected from 238 patients. Among those patients, 73 patients were survived and 165 patients were died.
