# Supplementary material for: Prevalence and clinical course of upper airway respiratory virus infection in critically ill patients with hematologic malignancies
Source: PLoS One. 2021 Dec 14;16(12):e0260741. doi: 10.1371/journal.pone.0260741 (PMC8670702; doi:10.1371/journal.pone.0260741)
Supplement: S7 Table — (DOCX) [file pone.0260741.s009.docx]

**S7 Table. Comparison of the clinical characteristics of hospital survivors and non-survivors in patients with positive upper airway respiratory virus PCR.**

| Variable | Survived patients  (n = 21) | Died patients  (n = 75) | *P* value |
| --- | --- | --- | --- |
| Age | 45.6 ± 16.8 | 55.7 ± 14.2 | 0.007 |
| Sex, Male | 16 (76.2) | 45 (60.0) | 0.269 |
| Allogenic HSCT recipients | 7 (33.3) | 30 (40.0) | 0.763 |
| Disease status |  |  |  |
| Active | 17 (81.0) | 63 (84.0) | 1.000 |
| Relapsed | 3 (14.3) | 26 (34.7) | 0.126 |
| SOFA score | 7.1 ± 3.6 | 10.7 ± 3.9 | <0.001 |
| Charlson Comorbidity Index | 2.0 (2.0 – 3.0) | 3.0 (2.0 – 5.0) | 0.029 |
| Nosocomial respiratory viral infection | 9 (45.0) | 43 (61.4) | 0.291 |
| Presence of pneumonia on ICU admission | 15 (71.4) | 69 (92.0) | 0.032 |
| Invasive pulmonary aspergillosis | 1 (4.8) | 9 (12.0) | 0.578 |
| Laboratory findings on ICU admission |  |  |  |
| Absolute neutrophil count, x 10^9^/L | 2.3 (0.6 – 6.8) | 2.5 (0.1 – 6.3) | 0.654 |
| Absolute lymphocyte count, x 10^9^/L | 0.3 (0.1 – 1.1) | 0.5 (0.1 – 1.5) | 0.329 |
| Procalcitonin, ng/mL | 2.9 (1.0 – 10.1) | 1.8 (0.9 – 4.8) | 0.472 |
| High sensitivity C-reactive protein, mg/dL | 16.1 ± 10.2 | 16.8 ± 9.2 | 0.762 |
| Reasons for ICU admission |  |  |  |
| Acute respiratory failure | 17 (81.0) | 68 (90.7) | 0.397 |
| Sepsis/Septic shock | 14 (66.7) | 56 (74.7) | 0.652 |
| Life-supporting interventions |  |  |  |
| High flow nasal cannula | 9 (42.9) | 33 (44.0) | 1.000 |
| Mechanical ventilation | 6 (28.6) | 65 (86.7) | <0.001 |
| Renal replacement therapy | 5 (23.8) | 23 (30.7) | 0.734 |
| Extracorporeal membrane oxygenation | 0 (0.0) | 2 (2.7) | 1.000 |
| Use of medications in 30 days prior to ICU admission |  |  |  |
| Use of corticosteroids | 20 (95.2) | 64 (85.3) | 0.401 |
| Accumulative prednisolone-equivalent dose | 10.8 (4.2 – 20.0) | 12.1 (4.2 – 21.8) | 0.696 |
| Use of immunosuppressants | 7 (33.3) | 21 (28.0) | 0.839 |
| Use of anti-cancer chemotherapeutic drugs | 15 (71.4) | 51 (68.0) | 0.973 |
| Other pathogens identified from respiratory specimens^a^ (n = 66) | 7 (46.7) | 28 (54.9) | 0.789 |
| Bacteria | 4 (26.7) | 20 (39.2) | 0.560 |
| Fungus | 3 (20.0) | 8 (15.7) | 1.000 |

Data are presented as number (percentage) or as median (interquartile range).

PCR, polymerase chain reaction; HSCT, hematopoietic stem cell transplant; SOFA, sequential organ failure assessment score; ICU, intensive care unit

^a^Appropriate lower respiratory tract specimen was collected from 66 patients. Among those patients, 15 patients were survived and 51 patients were died.
