# Supplementary material for: Prevalence and clinical course of upper airway respiratory virus infection in critically ill patients with hematologic malignancies
Source: PLoS One. 2021 Dec 14;16(12):e0260741. doi: 10.1371/journal.pone.0260741 (PMC8670702; doi:10.1371/journal.pone.0260741)
Supplement: S8 Table — (DOCX) [file pone.0260741.s010.docx]

**S8 Table. Multivariate analyses with logistic regression models for probability of in-hospital mortality in patients with positive upper airway respiratory virus PCR**

| Variable | Multivariable | | |
| --- | --- | --- | --- |
|  | Adjusted OR | 95% CI | *P* value |
| Age | 1.01 | 0.99 – 1.04 | 0.292 |
| SOFA score | 1.20 | 1.11 – 1.31 | <0.001 |
| Charlson Comorbidity Index | 1.03 | 0.86 – 1.24 | 0.754 |
| Presence of pneumonia at ICU admission | 4.90 | 2.33 – 10.29 | <0.001 |
| Mechanical ventilation | 6.86 | 3.81 – 12.36 | <0.001 |

PCR, polymerase chain reaction; OR, odds ratio; CI, confidence interval; SOFA, sequential organ failure assessment score; ICU, intensive care unit
